# Transcriptomic analysis of wheat near-isogenic lines identifies *PM19-A1* and *A2* as candidates for a major dormancy QTL

**DOI:** 10.1186/s13059-015-0665-6

**Published:** 2015-05-12

**Authors:** Jose M. Barrero, Colin Cavanagh, Klara L. Verbyla, Josquin F.G. Tibbits, Arunas P. Verbyla, B. Emma Huang, Garry M. Rosewarne, Stuart Stephen, Penghao Wang, Alex Whan, Philippe Rigault, Matthew J. Hayden, Frank Gubler

**Affiliations:** CSIRO Agriculture Flagship, GPO Box 1600, Canberra, ACT 2601 Australia; CSIRO Digital Productivity & Services Flagship, GPO Box 664, Canberra, ACT 2601 Australia; CSIRO Digital Productivity & Services Flagship, GPO Box 780, Atherton, QLD 4883 Australia; CSIRO Digital Productivity & Services Flagship, GPO Box 2583, Brisbane, QLD 4001 Australia; Department of Environment and Primary Industries, Agriobio Center, Bundoora, VIC 3083 Australia; Gydle, 101-1332 Av. Chanoine Morel, Québec, QC G1S 4B4 Canada; Current address: Bayer CropScience, Technologiepark 38, 9052 Zwijnaarde (Gent), Belgium; Current address: Department of Environment and Primary Industries, 110 Natimuk Rd, Horsham, VIC 3400 Australia

## Abstract

**Background:**

Next-generation sequencing technologies provide new opportunities to identify the genetic components responsible for trait variation. However, in species with large polyploid genomes, such as bread wheat, the ability to rapidly identify genes underlying quantitative trait loci (QTL) remains non-trivial. To overcome this, we introduce a novel pipeline that analyses, by RNA-sequencing, multiple near-isogenic lines segregating for a targeted QTL.

**Results:**

We use this approach to characterize a major and widely utilized seed dormancy QTL located on chromosome 4AL. It exploits the power and mapping resolution afforded by large multi-parent mapping populations, whilst reducing complexity by using multi-allelic contrasts at the targeted QTL region. Our approach identifies two adjacent candidate genes within the QTL region belonging to the *ABA-induced Wheat Plasma Membrane 19* family. One of them, *PM19-A1*, is highly expressed during grain maturation in dormant genotypes. The second, *PM19-A2*, shows changes in sequence causing several amino acid alterations between dormant and non-dormant genotypes. We confirm that *PM19* genes are positive regulators of seed dormancy.

**Conclusions:**

The efficient identification of these strong candidates demonstrates the utility of our transcriptomic pipeline for rapid QTL to gene mapping. By using this approach we are able to provide a comprehensive genetic analysis of the major source of grain dormancy in wheat. Further analysis across a diverse panel of bread and durum wheats indicates that this important dormancy QTL predates hexaploid wheat. The use of these genes by wheat breeders could assist in the elimination of pre-harvest sprouting in wheat.

**Electronic supplementary material:**

The online version of this article (doi:10.1186/s13059-015-0665-6) contains supplementary material, which is available to authorized users.

## Background

Seed dormancy is an adaptive trait in plants that imposes a temporal block on germination even under apparent favorable conditions. It evolved to optimize seed survival by avoiding germination under non-optimal environmental conditions. Depth and duration of dormancy are largely regulated by genetic and environmental factors, with temperature during seed development having a major role in determining dormancy acquisition [1]. Many crop plants exhibit weak dormancy as a result of the selection for rapid and uniform germination to maximize synchronization of crop production. In many instances this has led to seeds that are prone to pre-harvest sprouting (PHS) following wet and cool conditions [1]. In cereals such as bread wheat (*Triticum aestivum* L.), PHS can cause large economic losses exceeding $US1 billion per year [2] due to adverse effects on grain quality and yield [1, 3]. As a result, identification of genes controlling cereal grain dormancy has become a major goal for breeders to eliminate the incidence of PHS in modern wheat cultivars. The introgression of dormancy-related quantitative trait loci (QTL) into commercial varieties still remains the principal strategy to provide protection against this major agronomic problem. Such QTL have been previously identified in wheat (reviewed in [1, 4]). Some are specific to populations whilst others have been identified across multiple populations, including the major QTL which is located on chromosome 4AL [1, 4]. This QTL can explain up to 40 % of the dormancy variability in some populations and, although it has been a major target for scientists and breeders over the past decade, its genetic nature has remained unknown [1].

The identification and mapping of QTL at high resolution has been accelerated by recent advances in the generation of genetic resources like Multi-parent Advanced Generation Inter-Cross (MAGIC) populations [5]. Similarly, next-generation sequencing technologies have led to an explosion in the amount of data available for gene identification projects. For example, since the release of the rice draft genome in 2000, the number of cloned QTL in this crop has increased exponentially [6]. However, significant challenges remain for identifying the genetic variants underlying QTL in crops that have large, polyploid and poorly sequenced genomes. Here, we present an approach that combines the use of a MAGIC population with RNA-sequencing to rapidly fine-map and identify candidate genes underlying QTL in wheat. The most powerful aspect of this approach is the ability to exploit the contrast between the multiple alleles carried by the MAGIC parents, which enables accurate detection of QTL-linked single nucleotide polymorphisms (SNPs) and of differential gene expression within a QTL region.

We have utilized a four-parent MAGIC population [7] to identify grain dormancy QTL in multiple environments. We report on the genetic analysis of the major QTL located on chromosome 4AL. Several heterogeneous inbred families (HIFs) [8] were generated from the mapping population, and used to develop multiple near-isogenic lines (NILs) to validate the QTL within each genetic background and for gene expression analysis during grain development. This method enabled the identification of two adjacent closely related candidate genes that displayed sequence and/or expression changes associated with the dormancy phenotype of the NILs. Those genes, which we called *PM19-A1* and *A2*, encode proteins of the abscisic acid (ABA)-induced Wheat Plasma Membrane 19 (AWPM19) family. The first one was highly expressed in dormant lines while the second had several sequence changes. We demonstrated that the expression of *PM19-A1* and *A2* is suppressed by high temperatures during grain maturation, an environmental condition that suppresses grain dormancy. Finally, by generating transgenic plants with altered *PM19* expression, we confirmed that these genes are positive regulators of grain dormancy in wheat.

## Results

### Quantitative trait loci mapping using a wheat MAGIC population

**Fig. 1:**
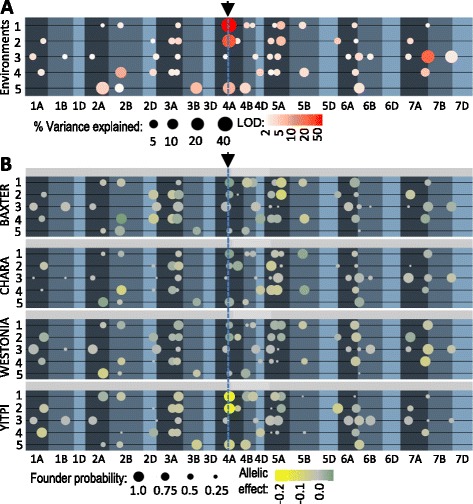
Location, size and founder effects of QTL for grain dormancy. Both panels show the environments on the vertical axis (1, glasshouse 2009 dark; 2, glasshouse 2009 light; 3, field 2009 dark; 4, field 2009 light; and 5, field 2010 dark), and genetic distance on the horizontal axis. Chromosomes are arranged linearly representing the entire genetic map, with 0 cM on the left for each chromosome. Background shading highlights boundaries between chromosomes and identifies genomes A, B and D going from darkest to lightest, respectively. **a** Dot size represents the percentage genetic variance explained for each QTL, and color intensity represents the statistical significance. Both size and color scales are log transformed to enable visualization of small effect QTL. **b** Founder contributions for each QTL. Each subpanel represents one of the four population parents. Dot size represents the likelihood of inheritance from that parent, color represents the direction of the allele effect on dormancy (yellow more dormant, green less dormant) and color intensity represents the size of the allele effect. The arrow and the vertical dotted line indicate the position of the QTL *4A-1*

A MAGIC population was developed by intercrossing four wheat varieties, Yitpi, Chara, Baxter and Westonia [7], and was used for mapping grain dormancy QTL. The population was used across three environments, one in glasshouse conditions in 2009 and the two other in the field in 2009 and 2010. Dormancy was measured by calculating the Germination Index (GI) of grain harvested at maturity (see Materials and methods). The grain harvested in 2009 was scored for dormancy under two different conditions, continuous light or darkness. This difference in conditions allowed us to better capture the dormancy phenotype of our material. In darkness grain dormancy is manifested with less strength than under light, so grain that retains high dormancy will show moderate GI in darkness but very low GI under light. On the contrary, grain that retains low dormancy will show a moderate GI under light but will show very high GI in darkness. For example, the grain harvested in 2010 was only scored in continuous light because even under that condition dormancy was low in comparison with previous screenings. Histograms of the GI for the five dormancy screenings are given in Additional file 1 and the mean GI for the parental lines for each screening can be found in Additional file 2. A linkage map was generated based on previously published Infinium iSelect SNP assays [9, 10] and utilized for QTL detection. Heritability across all site/year combinations ranged from 0.11 to 0.56 (Additional file 2). In total, 54 QTL were identified across all datasets (Fig. 1a) that, based on their map locations and the pattern of effect sizes from each of the four founders, could represent 39 unique QTL (Additional file 3).

**Fig. 2:**
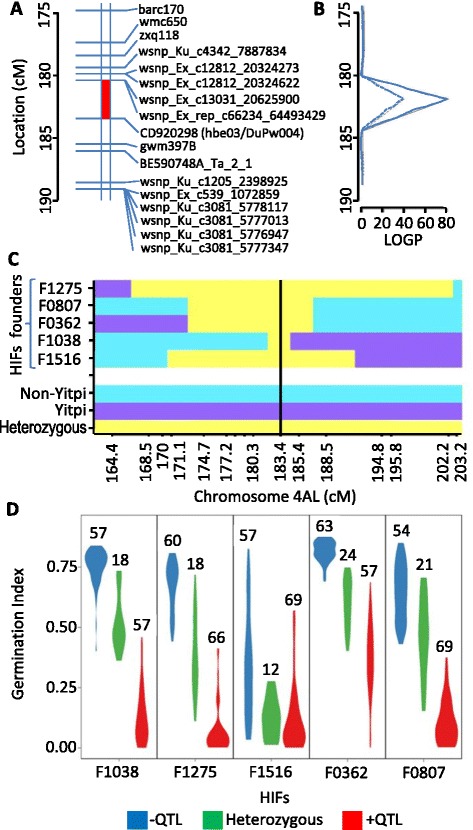
Map of the *4A-1* QTL region, genotype of the individuals selected as HIF founders and phenotype of the HIFs. **a** Genetic map of the *4A-1* QTL region. The red section indicates the highest probability interval for the QTL. **b** Trace of the log probabilities (LOGP) for this region for the dark (solid line) and light (dashed line) glasshouse data. **c** Illustration of the heterozygous regions in the HIF founders. **d** Illustration of the germination phenotype (GI) of the progeny of the five HIFs. The colors represent the frequency of the different genotypes: homozygous plants with the QTL (red), homozygous without the QTL (blue) and heterozygous (green). The areas representing the different classes are equivalent and the number of individuals tested within each class is given

The major QTL detected, named *4A-1*, explained between 5 and 40 % of the genetic variance and was located on the long arm of chromosome 4 (Fig. 1a). This QTL was mapped between the markers wsnp_Ex_c66324_64493429 and CD920298 (Fig. 2b). In that QTL, the allele carried by Yitpi contributed to high dormancy, while the alleles carried by the other cultivars conferred less dormancy (Fig. 1b). We did not detect the *4A-1* QTL in the 2009 field trial (Fig. 1a), possibly due to the strong influence of environmental conditions on the genetic control of dormancy. This QTL was targeted for further genetic analysis.

### Generation of multiple near-isogenic lines and RNA-seq analysis

We generated multiple NILs segregating for the *4A-1* QTL following a method based on HIFs analysis [8]. Five F_7_ RILs from the mapping population were chosen as founders of HIFs on the basis of being heterozygote for the *4A-1* QTL region (Fig. 2c), while maximizing homozygosity for the background genome (about 97 % homozygosis). From them, five F_8_ HIFs were obtained by self-pollination and were phenotyped and genotyped. In every HIF, homozygote individuals for the Yitpi allele showed low GI while homozygous for the other alleles showed high GI. Heterozygous individuals showed intermediate GI values in at least four out of the five HIFs (Fig. 2d). From each HIF, homozygous individuals with contrasting QTL effects were selected and self-pollinated to generate five F_9_ sets of NILs. Those NILs were finally grown together with the four parent varieties, and grain samples collected at 15, 25 and 35 days post-anthesis (DPA). RNA was isolated from those samples and pooled together in two groups, one containing all samples coming from NILs carrying the QTL (pool A) and the other containing all samples coming from NILs without the QTL (pool B). Samples collected from the four parents at the same DPA were also included in the corresponding RNA pool. This innovative pooling approach allowed us to distinguish a real signal coming from the targeted selected region (segregating equally in each set of NILs) while background noise coming from the rest of the genome is neutralized (different in every set of NILs).

Using the International Wheat Genome Sequencing Consortium (IWGSC) flow-sorted Chromosome Survey Sequence (CSS) contigs of Chinese Spring [11] as a reference, the pooled sequence reads were mapped as described in Materials and methods. Differential expression analysis based on mapped reads identified a total of 39 differentially expressed CSS contigs (Additional file 4A), four of them on the 4AL chromosome. Only one of them, Chr4AL_7123764, was within the *4A-1* QTL region (CSS contigs were mapped to the QTL region by directly aligning them against the physical QTL supercontig sequence described below in the ‘Physical mapping’ section) and that contig was ranked first based on statistical significance. Furthermore, SNP analysis between the two pools identified thirteen SNPs in seven CSS contigs, all on chromosome 4AL (Additional file 5). In three of those contigs the SNPs caused an amino acid change in an annotated gene (Additional file 5), and only two of them were mapped to the QTL region: Chr4AL_7123764 (which was also differently expressed) and Chr4AL_7174272. Both of these contigs contained a gene that belonged to the same family and these were chosen as the best candidates.

### Description of the candidate genes *PM19-A1* and *A2*

**Fig. 3:**
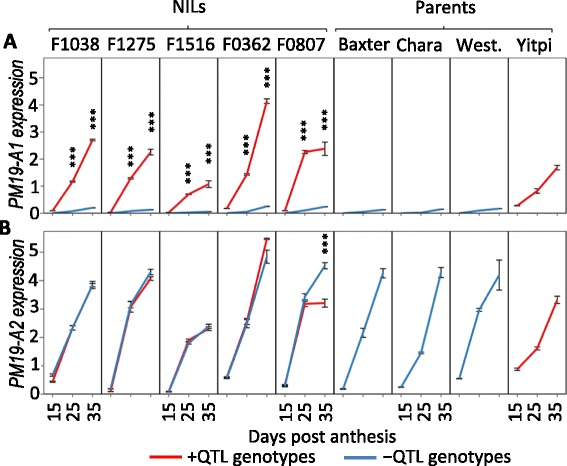
Gene expression profile of *PM19-A1* and *PM19-A2* in NILs and parental lines. qPCR analysis of the expression of the candidate genes *PM19-A1* (**a**) and *PM19-A2* (**b**) in each set of NILs segregating for the QTL and in the parental lines. Expression was analyzed during grain development at three time points: 15, 25 and 35 DPA. The average of three biological replicates is shown with the standard error. Statistically significant differences using *T*-test are indicated with asterisks (*p < 0.1; **p < 0.05; ***p < 0.01)

**Fig. 4:**
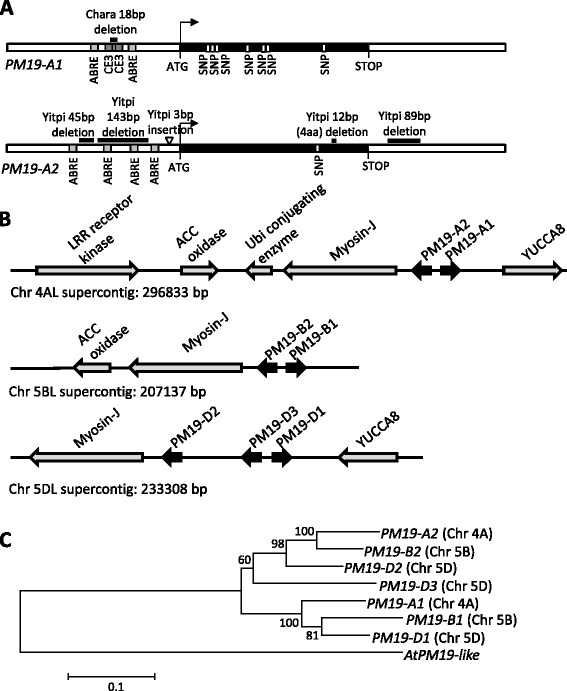
Characterization of the *PM19* gene family. **a** Gene models of the *PM19* genes in the QTL region, *PM19-A1* and *PM19-A2*, showing in black the coding regions. Grey boxes in the promoters indicate the position of ABA-related motifs (ABRE and CE3 elements). Deletions and insertions between the two haplotypes identified for each gene are indicated above the genes by black bars or by a triangle, respectively. **b** Schematic representation of the homeologous supercontigs containing the *PM19* genes. The arrows indicate the position of the genes. The *PM19* genes are highlighted in black. **c** Phylogenetic tree of the *PM19* gene family in wheat. The sequence of the *Arabidopsis* orthologue gene *AtPM19-like* was use as outlier. Numbers indicate the bootstrap support

Chr4AL_7123764 contained a single gene with similarity to a gene encoding the AWPM19 protein [12], which we named *PM19-A1*. This gene was highly expressed in NILs carrying the QTL and in the dormant parent, and had low expression in non-dormant genotypes (Additional file 4). *PM19-A1* expression increased with grain maturation, being low at 15 DPA and reaching its maximum at 35 DPA (Additional file 4B). The RNA-seq results were successfully validated by quantitative PCR (qPCR; Fig. 3a). We also identified several SNPs in this gene in the RNA-seq analysis that we confirmed by Sanger sequencing. We identified two allelic variants differentiated by seven SNPs, of which three caused amino acid changes between the dormant and the other parents. An 18-bp deletion was also identified in the promoter regions of the parents without the QTL, but not in Yitpi (Fig. 4a). We developed a set of primers (Additional file 6) spanning the deletion that can be used as a genetic marker (the marker product size being 117 bp for the QTL donor and 99 bp in the other founders).

Chr4AL_7174272 contained a second gene from the AWPM19 family that we named *PM19-A2.* We sequenced *PM19-A2* in the parents of the MAGIC population and found that the dormant allele carried by Yitpi had several deletions in the promoter region and an 89-bp deletion on the 3′ untranslated region compared with the other cultivars. We also found a single non-synonymous SNP and a 12-bp in-frame deletion causing the loss of four amino acids (Fig. 4a). The transcriptome analysis did not show any expression difference for this gene, and this was confirmed by qPCR (Fig. 3b).

**Fig. 5:**
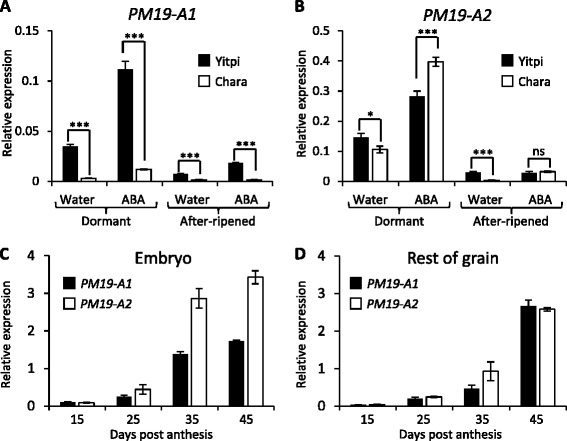
Expression of *PM19-A1* and *PM19-A2* in grains during development and in response to ABA after imbibition. qPCR analysis of the expression of *PM19-A1* (**a**) and *PM19-A2* (**b**) in embryos isolated from mature dormant and after-ripened grain of Yitpi and Chara imbibed for 24 h in water or in 10^−4^ M ABA. Expression of *PM19-A1* and *PM19-A2* in embryos (**c**) and in rest-of-grain (grain coats and endosperm) samples (**d**) of the Yitpi parental line dissected during grain development at four time points: 15, 25, 35 and 45 DPA. The average of three biological replicates is shown with the standard error. Statistically significant differences using *T*-test are indicated with asterisks (*p < 0.1; **p < 0.05; ***p < 0.01; ns, non-significant)

The polymorphisms found in the promoters of both *PM19-A1* and *A2* affected several motifs related to the responsiveness to ABA, a key plant hormone involved in dormancy control [3]. The deletion in the *PM19-A1* promoter in Chara altered the spacing between two ABA-response elements (ABREs) and two coupling elements (CE3), and the deletion in the *PM19-A2* promoter in Yitpi affects several ABRE motifs (Fig. 4a). In order to study the expression of *PM19-A1* and *A2* in response to ABA, dormant (freshly harvested) and 6 months after-ripened Yitpi and Chara grains were imbibed for 24 h in water or in ABA, and embryos were isolated for qPCR analysis. *PM19-A1* was, as expected, highly expressed in Yitpi dormant grains while Chara showed very low expression (Fig. 5a). After ABA treatment the expression of *PM19-A1* was again much higher in Yitpi than in Chara, although the expression increased about four fold in both genotypes (Fig. 5a). *PM19-A2* expression was also higher in Yipti dormant grain imbibed in water, but differences were small compared with those found for *PM19-A1* (Fig. 5b). After the ABA treatment, *PM19-A2* was also induced in the two wheat cultivars. In Yitpi, both genes were clearly more highly expressed in dormant grains than in after-ripened grains, but in Chara, *PM19-A1* showed very low expression in both dormant and after-ripened grains (Fig. 5a,b).

To further characterize the expression pattern of the candidate genes during grain development, embryo samples were dissected from the Yitpi parental line at 15, 25, 35 and 45 DPA, and used for qPCR analysis (Fig. 5c). De-embryonated grain samples were also kept and used to quantify gene expression (Fig. 5d). Both *PM19-A1* and *PM19-A2* were expressed in embryo and rest-of-grain (grain coats plus endosperm). Both genes followed a similar expression trend in all samples, with increasing expression following maturation and reaching a maximum expression at 45 DPA. By 35 DPA, gene expression in embryos was close to maximum, while expression in the rest-of-grain was still low. At all stages, *PM19-A2* showed higher expression than *PM19-A1* in embryos, while both genes showed similar gene expression in de-embryonated grains.

### Physical mapping and gene family characterization

To characterize the region surrounding our candidate genes, a Chinese Spring wheat bacterial artificial chromosome (BAC) library [13] was screened with the markers encompassing our QTL region. We also screened the BAC library using primers from a conserved *PM19* region in order to identify BACs carrying *PM19* genes. Seven BACs (see Materials and methods) were identified as positive for one of several of our markers. Those BACs were sequenced and assembled into three supercontigs (see Materials and methods). Each one spanned multiple mapped CSS contigs (Additional file 7) which were used to assign the supercontigs to the chromosomes 4AL, 5BL and 5DL. BLAST-based searches identified several genes within each supercontig (Fig. 4b). In the supercontig mapped to chromosome 4AL, we found the gene *PM19-A1* next to *PM19-A2*. In addition, we identified genes encoding a myosin, an ACC-like oxidase, a Yucca protein, a ubiquitin conjugating enzyme and a leucine-rich repeat receptor kinase, all of which were expressed at low levels in our transcriptomic data and none of which were found differentially expressed or with polymorphisms causing amino acid changes. The markers that flanked the interval with the highest probability to contain the *4A-1* QTL were found inside the 4AL supercontig for which we have the whole physical sequence (the marker wsnp_Ex_c66324_64493429 is in the leucine-rich repeat receptor kinase and the marker CD920298 is in *PM19-A2*).

In the 5BL supercontig two *PM19* genes were found (named *PM19-B1* and *B2*) along with genes encoding a myosin and an ACC-like oxidase. In the 5DL supercontig three *PM19* genes were identified (named *PM19-D1*, *D2* and *D3*) as well as genes encoding a myosin and a Yucca protein. The presence of almost the same pattern of genes in the three supercontigs indicates that they are homeologous regions. This was supported by the phylogenetic analysis of the seven *PM19* genes identified, clearly highlighting the existence of two clades, each with a copy on the 4AL, 5BL and 5DL chromosomes (Fig. 4c). The third *PM19* gene on chromosome 5DL may have arisen from a more recent duplication. The *PM19* genes located on chromosomes 5BL and 5DL were found to be expressed at low levels in our transcriptomic data experiment, but none was differentially expressed between the pools.

In addition, we sequenced amplicons associated with the markers that were linked to the *4A-1* QTL. We found that the markers hbe03 and DuPw004 [14, 15] (Fig. 2a) spanned the same polymorphism (an INDEL of 89 bp). We also found that they were part of an expressed sequence tag (EST CD920298) previously mapped to the 4AL QTL region [15]. Based on the 4AL supercontig, we found that this EST was derived from the gene that we named *PM19-A2*. Apart from that, we also found that the originally described AWPM19 protein is encoded by the *PM19-D3* gene on chromosome 5D. This gene was first named *WPM-1* [12] and more recently *TaPM19-1* [16]. We did not find any polymorphism in this gene between the MAGIC parents.

### Generation of wheat transgenic lines silencing *PM19* genes

**Fig. 6:**
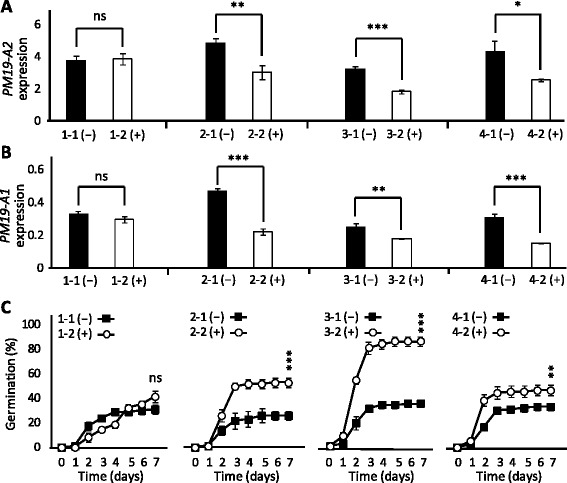
Gene expression analysis and dormancy phenotype of the RNAi transgenic plants. Four independent transgenic homozygous lines carrying a hairpin construct against *PM19-A2*, with their corresponding null-segregants, were tested for expression of *PM19-A2* (**a**) and *PM19-A1* (**b**), and for dormancy (**c**). Null-segregants are shown in black and homozygote transgenic plants are shown in white. The average of three replicates (five for the dormancy assay) is shown with the standard error. Statistically significant differences using *T*-test are indicated with asterisks (*p < 0.1; **p < 0.05; ***p < 0.01; ns, non-significant)

To functionally test the role of *PM19* genes on grain dormancy, we used an RNA interference (RNAi) approach in the transformable cultivar ‘Bob White 26’. This cultivar is genetically similar to Chara, Westonia and Baxter, having a low *PM19-A1* expression but high *PM19-A2* expression. For that reason the RNAi hairpin construct was designed using the *PM19-A2* sequence in order to silence that gene. Four independent homozygous transgenic T_3_ lines, with their corresponding null-segregants, were generated and harvested at maturity for gene expression and dormancy analysis. Three of the four transgenic lines showed silencing of the targeted gene in comparison to the controls (Fig. 6a). Given that our RNAi approach would likely target other *PM19* genes, we also tested the effect of the hairpin on the expression of *PM19-A1*, and although it was expressed at a very low level in the control plants, the expression was even lower in the transgenics (Fig. 6b). Dormancy assays revealed a decrease in grain dormancy for lines 2, 3 and 4, while no change was detected in line 1 (Fig. 6c). These results confirm that the *PM19* genes are positive regulators of grain dormancy.

### Effect of temperature on *4A-1* quantitative trait locus expression

**Fig. 7:**
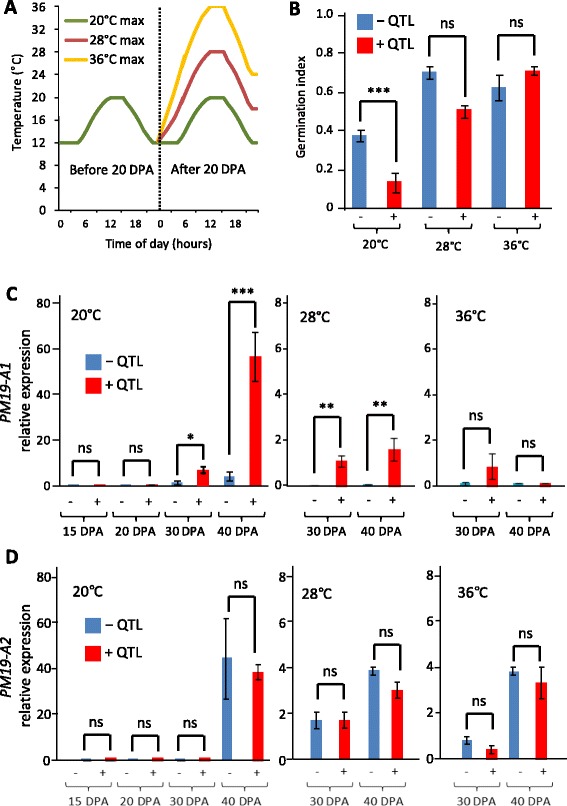
Temperature effects on the expression of the *4A-1* QTL. NILs derived from one of the HIFs were grown under three temperature regimes after 20 DPA (**a**). Grain was harvested at maturity for dormancy tests (**b**) and during grain development for expression analysis of *PM19-A1* (**c**) and *PM19-A2* (**d**). The average of three replicates is shown with the standard error. Statistically significant differences using *T*-test are indicated with asterisks (*p < 0.1; **p < 0.05; ***p < 0.01; ns, non-significant)

Temperature is one of the key environmental factors affecting dormancy development [1]. We designed an experiment to test if high temperatures during late grain maturation could suppress the expression of the *4A-1* QTL. One of the NIL sets was grown in cabinets under three temperature regimes applied after 20 DPA, reaching 20 (control), 28 or 36 °C at the middle of the day (Fig. 7a). Grains were harvested at maturity and germination scored. At 20 °C, the genotype carrying the QTL showed higher dormancy than the genotype without the QTL. At 28 °C, both genotypes displayed a loss of dormancy and the QTL effect was minimal. At 36 °C, the presence or absence of the QTL had no effect (Fig. 7b). We studied the expression of *PM19-A1* and *A2* in grains harvested at different developmental stages from the three temperature conditions. As expected, *PM19-A1* expression was highly induced after 30 DPA in the genotype carrying the QTL and the difference in expression was maximal at 40 DPA. The expression of this gene was strongly suppressed by increasing temperature, being about tenfold lower in the 28 °C treatment and completely suppressed at 40 DPA in the 36 °C treatment (Fig. 7c). Also as expected, there were no differences in the expression of *PM19-A2* between genotypes and the expression of this gene was also maximal at 40 DPA in the control treatment. Higher temperatures also inhibited the expression of *PM19-A2* and both 28 °C and 36 °C treatments reduced its expression by about tenfold (Fig. 7d) in both genotypes.

### *PM19-A1* and *A2* haplotype analysis and expression across diverse wheat varieties

**Fig. 8:**
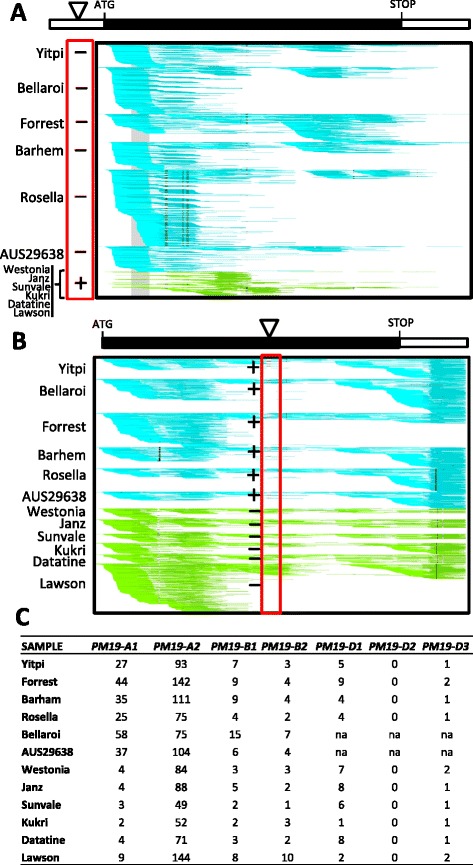
Expression profiles and genotypes for *PM19-A1* and *A2* across 12 wheat varieties. RNA-seq data were aligned to the seven wheat *PM19* genes. **a** For *PM19-A1* two distinct expression patterns were identified across the varieties tested, one showing high expression (Yitpi-like, blue reads) and the other showing low expression (Westonia-like, green reads). **b** For *PM19-A2* no expression differences were found. Wheat variety names are shown to the left of the panels. For *PM19-A1* the presence (plus sign) or absence (minus sign) of the 18-bp promoter deletion is indicated. For *PM19-A2* the presence (plus sign) or absence (minus sign) of the 12-bp in-frame deletion is indicated. The approximate position of these two polymorphisms, which showed no recombination in any variety, is indicated with a triangle. Other segregating polymorphisms (black vertical lines) are visible across the panels and not linked to the *PM19-A1* expression phenotype. **c** Summary of the expression results for the seven *PM19* genes across all the varieties. The numbers indicate counts per million reads. The differences in *PM19-A1* expression between the genotypes with and without the 18-bp promoter deletion were highly significant (*T*-test *P*-value = 6.1E-05). No significant differences in the expression were found for the other genes

In order to evaluate the correlation between the deletion on the promoter of *PM19-A1* and the gene expression of the gene, we analyzed 12 genetically diverse wheat varieties. The 12 varieties included Yitpi and Westonia as haplotype controls, and a durum (Bellaroi) and a synthetic hexaploid (AUS29638; derived by crossing durum wheat with a diploid ancestor of bread wheat, *Aegilops tauschii* Coss) varieties. Transcriptomic analysis showed that the expression levels of *PM19-A1* were conserved across wheat, with the six lines with high expression having the Yitpi-like promoter, and the six with low expression having the 18-bp promoter deletion (Fig. 8a, c). Interestingly, the durum and synthetic lines both had Yitpi-like haplotype and expression of *PM19-A1*. We also analyzed the expression of *PM19-A2* and this gene showed similar expression levels in all varieties (Fig. 8b, c). Genotyping across the 12 varieties showed considerable polymorphism across the genes *PM19-A1* and *A2*. Only the *PM19-A2* 12-bp in-frame deletion and the *PM19-A1* 18-bp promoter deletion were completely associated in the manner observed in the mapping population (Yitpi having the full *PM19-A1* promoter and the 12-bp deletion in the *PM19-A2* open reading frame, and the other parental lines having the 18-bp deletion in the *PM19-A1* promoter and no deletion in the *PM19-A2* gene). Amongst the 12 lines, no recombinants were found between these two polymorphisms despite evidence of extensive historical recombination of surrounding polymorphisms. We also analyzed the expression of the other *PM19* genes*.* In all varieties, the expression of the genes located in chromosomes 5BL and 5D was clearly much lower than the expression of the genes located in chromosome 4AL (Fig. 8c), which suggests *PM19-A1* and *A2* have a more important contribution to dormancy.

## Discussion

Using a wheat MAGIC population, we have identified several dormancy QTL and have targeted for genetic analysis the major one, which is located on chromosome 4AL. A QTL in the same region has been previously reported in other bi-parental populations as a major source of dormancy [1, 4]. We utilized an allelic series of HIFs chosen so that historical recombinants were tiled across the target *4A-1* QTL site. These HIFs were used to develop five independent sets of NILs that were analyzed in an innovative transcriptomic pool comparison to identify SNPs and differentially expressed sequences in the QTL region. A similar multi-NIL transcriptomic approach has been suggested previously as a method for detecting candidate genes for QTL [17], but to our knowledge this is the first attempt to use it successfully. Our NILs are derived from a MAGIC population, and consequently a greater number of alleles are segregating at any given locus, which allows a more robust contrast analysis. This pipeline revealed *PM19-A1*, a newly identified gene whose high expression during late grain maturation is associated with the dormant genotypes. We also identified a second gene, *PM19-A2*, which showed changes in sequence between the genotypes segregating for the QTL. In addition we identified several differentially expressed genes that did not map to the QTL region but to other locations. Those could be part of the downstream transcriptional response of the targeted QTL [17], and their putative functions are being investigated.

Two allelic variants for *PM19-A1* and *A2* were identified in the founders of our mapping population. In each case, the dormant allele differed from those of the other three founders. Several polymorphisms were found in each gene and we observed an intermediate dormancy phenotype in the heterozygous individuals from the HIFs, which demonstrates the semi-dominant inheritance of this QTL. A reduction in dormancy in independent transgenic lines having reduced *PM19* expression directly links the dormancy phenotype with the activity of these genes. In the temperature study, we found differences in expression of *PM19-A1* and these were highly correlated with the loss of dormancy with increasing temperature. These results support that *PM19-A1* expression could explain the QTL and we propose that the deletion in the promoter of *PM19-A1* is the most likely cause of the expression variation observed. This deletion affects the spacing between two ABRE motifs and two CE3 motifs (Fig. 4a), which are well-characterized motifs involved in responsiveness to ABA, a key dormancy-promoting factor [18]. The spacing between those motifs is critical for ABA responsiveness [19]. Analysis of a panel of wheat varieties showed that the *PM19-A1* expression profiles observed in the parental lines of our mapping population were also found across genetically diverse varieties. The two wheat lines carrying a 4A chromosome from durum (Bellaroi and AUS29638) had high expression (Yitpi-like) of *PM19-A1*, suggesting the expression pattern predates the formation of hexaploid bread wheat about 10,000 years ago. All the other varieties tested that had the Yitpi promoter haplotype also displayed high *PM19-A1* expression. The six varieties tested that had Chara-like promoters all had low gene expression. Interestingly, the existence of the low-expression promoter in Australian wheat varieties also hints that the non-dormant-type allele may have previously been a target of positive selection during domestication, although this remains to be tested. In relation to *PM19-A2*, we found that the 12-bp deletion in the coding region was always completely linked to the *PM19-A1* 18-bp promoter deletion in all varieties, despite extensive sharing of polymorphisms at nearby markers. This evidence for historical recombination between *PM19-A1* and *A2* but retention of the two deletions in coupling may indicate that these two variants operate epistatically to cause the QTL phenotype. The co-inheritance of these two polymorphisms across such diverse material also suggests it will be difficult to find lines in which the independent effects of these two polymorphisms can be tested.

The temperature effect on the expression of this gene provides an explanation of why the *4A-1* QTL was not detected in the 2009 field trial. Changes in temperature during late maturation have been linked to the suppression of dormancy and PHS incidence [20, 21] and have a critical effect on the expression of the QTL [22]. In agreement with that, 2009 was the hottest year in the last 10 years at the location where our field experiments were performed (see Materials and methods). While expression of *PM19-A2* was also temperature regulated, we did not detect clear differences between genotypes or in the responsiveness of this gene to ABA or to after-ripening between the wheat varieties. This indicates that the polymorphisms we identified in the promoter of *PM19-A2* have no effect on the dormancy behavior (even when some ABREs are affected). However, we cannot rule out that the four amino acid difference in PM19-A2 between Yitpi and the non-dormant cultivars could have an impact. As we discussed in the previous paragraph, it is also very likely that the combination of both changes in those genes could be the reason for the detection of this QTL. In any case, the new gene-based markers identified here will improve the accuracy of the QTL selection and remove the current risk of misclassification in breeding programs.

Apart from *PM19-A1* and *A2*, we identified five other genes within the *PM19* family, which we named *PM19-B1*, *B2*, *D1*, *D2* and *D3* (previously named *AWP-1* and *TaPM19-1*) [12, 17]. The genes *PM19-A1* and *A2* are located in the centromeric region of chromosome arm 4AL, while *PM19-B1* and *B2* are located on the telomeric region of chromosome 5BL and *PM19-D1*, *D2* and *D3* on the telomeric region of 5DL. These locations can be explained by the ancestral translocation that occurred between chromosome 4A and 5A [23, 24] and implies that the *4A-1* QTL was translocated in that event from chromosome 5AL. The *PM19* orthologues from *Brachypodium* (Bradi1g00600) and barley (AF218627) [25] are found in the telomeric region of chromosomes 1 and 5HL, respectively, which further supports the *4A-1* translocation hypothesis. The barley *PM19* orthologue was shown to be embryo-specific, with expression that decreased upon germination but stayed high in dormant embryos [25]. Very interestingly, a major dormancy/malting QTL (named SD2) in barley located in the telomeric region of chromosome 5HL [26] is thought to be syntenic with the wheat 4AL QTL [23, 27]. In *Arabidopsis*, the orthologue gene *AWPM19-like* (At1g04560) maps inside the region associated with the seed dormancy QTL *Delay of Germination 2* [28], which makes it a good candidate for that QTL in this species as well.

The seven wheat *PM19* genes described in this work show great similarity at both the nucleotide and amino acid levels (over 90 % protein identity), which could suggest that they all have a role as dormancy promoters. However, our expression results indicate that the expression of *PM19-A1* and *A2* is clearly higher in comparison with the others, suggesting that the genes on chromosome 4AL may make a bigger contribution. Analysis of the AWPM19 protein sequence showed it is highly hydrophobic and it has four putative membrane-spanning domains. Similarities between AWPM19 and the soybean *GmPM3* gene product were identified, indicating that AWPM19 is conserved and exist across a wide range of higher plant species [12]. Members of the *PM19* gene family have been associated with ABA action. The first description of the AWPM19 protein, encoded by the *AWP-1* gene on chromosome 5D, showed that increased freezing tolerance of ABA-treated wheat suspension-cells was strongly associated with the accumulation of this protein [12]. More recently, this gene has been shown to be highly expressed in grain during late maturation and to be induced by ABA in the roots [16]. Our results demonstrate also that our candidate genes *PM19-A1* and *A2* are highly expressed in grains during late maturation in both embryo and de-embryonated grain. We have also proven that these genes are strongly induced by ABA in imbibed grains, thus supporting their role as dormancy promoters. In agreement with that, we have also shown that, in Yitpi, both *PM19-A1* and *A2* are more highly expressed in dormant than in after-ripened grains.

Apart from being related to ABA, the function of PM19 protein and the mechanism by which it regulates dormancy are unknown. Previous studies in wheat indicated that PM19 is localized in the plasma membrane [12], and other indirect studies in *Arabidopsis* identified it in the seed oil bodies [29]. These findings indicate that PM19 could be part of a regulatory pathway linking ABA with the membrane/lipidome. Recently, a strong gene candidate was proposed for another major wheat dormancy QTL located on chromosome 3AS [30, 31]. That gene, named *MOTHER OF FT AND TFL1* (*MFT*), belongs to a family of genes encoding the phosphatidylethanolamine-binding proteins and is involved in dormancy acquisition during grain maturation in response to temperature [30]. Phosphatidylethanolamine is an abundant membrane phospholipid in seeds. Both the *PM19* genes and *MFT* share several similarities: their effects on grain dormancy are affected by the temperature in which the grain develops and their functions could be linked to the plasma membrane. Future studies will address if these two classes of genes interact or have independent functions, and also will investigate if the different *PM19* genes have similar or distinct roles during dormancy acquisition and germination.

## Conclusions

Increasing grain dormancy in wheat is an important goal for breeders globally in order to reduce the risk of pre-harvest sprouting. However, the identification of dormancy-related genes has been hampered by the complexity of the wheat genome. We have established a new pipeline for cloning QTL in wheat, and we have demonstrated its use by applying it to target the major grain dormancy QTL located on chromosome 4AL. Using this approach we have identified in the QTL region two adjacent candidate genes of the same family displaying sequence and expression changes. Functional analysis revealed that those genes, named *PM19-A1* and *PM19-A2*, are positive regulators of dormancy in wheat. The new gene-based markers identified here will improve the accuracy of QTL selection in breeding programs. Our multi-NIL transcriptomic analysis provides a new QTL-to-gene method for the rapid identification of both candidate genes and new markers for fine mapping.

## Materials and methods

### Population and genetic map construction

A four-way MAGIC population consisting of 1579 F_7_ RILs developed by intercrossing four Australian wheat varieties [7] was used for this study. MAGIC populations are constructed using a set mating design where inter-crosses of (*2*^*n*^) diverse founders are crossed for *n* generations and then selfed for a number of generations to obtain RILs. Due to such a crossing structure, across the genome of each line there is an equal chance of contributions from every founder. The population used for this study was constructed using the four Australian commercial cultivars Baxter, Chara, Westonia and Yitpi.

The genetic data consisted of 5763 markers, mostly SNPs [9], with the addition of 39 multi-allelic microsatellites and about 800 DArT markers [7]. The map included 21 linkage groups corresponding to the 21 hexaploid wheat chromosomes, and three additional groups of markers for which chromosome assignment could not confidently be assigned. Co-located markers (at the same position on the map) were removed prior to QTL analysis. The map used for QTL analysis had a total length of 5788 cM and consisted of 3230 markers.

### Glasshouse and field trials

One glasshouse and two field trials were performed. Partially replicated designs were used for all trials and were spatially optimized [32]; DiGGer [33] was used to generate the designs. Each trial contained a set of check cultivars which always included the parental lines from the four-way MAGIC population.

The glasshouse trial was carried out in 2009 in Canberra, New South Wales, Australia. There were ten blocks each consisting of four benches, with each bench laid out as 12 rows by 5 columns, so that a block consisted of 48 rows by 20 columns. The trial included 1557 four-way MAGIC F_7_ lines with partial replication of 20 % and at least seven replicates of the four check cultivars.

The first field trial was conducted in 2009 at Yanco, New South Wales, Australia. The trial was laid out in 81 rows by 20 columns in 3 blocks of 27 rows by 20 columns, and consisted of 1100 F_6:8_ RILs from the four-way MAGIC population and 16 check cultivars. Forty percent of the RILs were replicated and the check cultivars were replicated a minimum of three times (three to seven).

A second field trial was conducted in 2010 at Leeton, New South Wales, Australia. Again a partially replicated design was used with 40 rows by 37 columns (2 blocks of 20 rows by 37 columns). The 1026 four-way MAGIC lines were partially replicated (40 %), with 14 check cultivars replicated at least twice (two to seven times).

At physiological maturity (using the collapse of the first node as a visual marker) two spikes (glasshouse) or four spikes (field) per plant were harvested and dried at 37 °C for 24 h. Spikes were then left at room temperature for 7 days, after which time they were stored at −20 °C.

Field trial weather conditions were recorded at the Yanco Agricultural Institute weather station, NSW, Australia (station number 074037; latitude 34.62°S, longitude 146.43°E) from the Australian Bureau of Meteorology [34]. The average maximum temperature in November, during grain maturation, was 33.2 °C in 2009 and 26.8 °C in 2010. The average minimum temperature in November was 18.3 °C in 2009 and 14.2 °C in 2010. The year 2009 was the hottest recorded in this region since 1999.

### Growth chamber experiments

For temperature analysis F_9_ NILs (with and without the 4AL QTL) derived from RIL F1038 were planted at the National Phenomics Facility in Canberra in three Conviron (Winnipeg, Manitoba, Canada) growth chambers (24 plants per chamber) in long day conditions (16 h light) and with a sine temperature regime reaching 20 °C at midday and 12 °C at midnight. At 20 DPA the temperature regime of one chamber was increased to reach 28 °C at midday and 18 °C at midnight, and in another chamber to reach 36 °C at midday and 24 °C at midnight. The third chamber was left with the original conditions as a control. Grains were harvested from the three cabinets at 15 and 20 DPA (before the temperature shift) and at 30 and 40 DPA (after temperature shift). Three biological samples of ten grains were collected per genotype and per temperature treatment. RNA was extracted from the samples and used for qPCR studies. Additional samples were collected at maturity for dormancy assays.

For the diversity analysis, 12 genetically diverse Australian wheat varieties, Yitpi (Australian hard wheat), Westonia (Australian premium white noodle wheat), Bellaroi (Durum wheat), Forrest (Australian premium white wheat), Barhem (Australian soft wheat), Rosella (Australian standard white noodle wheat), AUS29638 (synthetic derivative), Janz (Australian hard wheat), Sunvale (Australian prime hard wheat), Kukri (Sponge and dough wheat), Datatine (Club-headed wheat) and Lawson (Red feed wheat), were grown in long day conditions in controlled environment chambers at the Victorian Agri-Biosciences Centre. Grain samples were collected at 32 DPA for RNAseq analysis of *PM19* gene expression.

### Dormancy screening

Prior to dormancy screening, spike samples were randomly allocated using a partially replicated design (with replication varying between 10 and 15 % across the trials) to batches consisting of approximately 180 samples for conducting the dormancy assays. Spikes were hand-threshed and 20 grains per replicate were placed on 90 mm Petri dishes containing one 90 mm Whatman 598 filter paper and 5 ml of water. The plates were sealed with parafilm and incubated at 20 °C under continuous white light at 130 μmol m^−2^ s^−1^ (Phillips TLD 36 W/865 fluorescent tubes) for the light treatment or wrapped in two layers of aluminum foil for dark treatment. Germination was scored daily for 7 days, counting grains with emerged coleorhiza as germinated. A weighted GI was calculated using the formula GI = (7 × N1 + 6 × N2 + … + 1 × N7)/(n days of test × Total grains) [35], where N1, N2, and N7 are the number of grains that had germinated on day 1, day 2 or day 7. The maximum GI is 1 if all grains germinate by day 1 (complete loss of dormancy), and the minimum is 0 if none germinated after 7 days (fully dormant).

The dormancy screen occurred over 10 weeks, 9 weeks or 6 weeks for the glasshouse, Yanco field trial and Leeton field trial, respectively. The screen was conducted for the two treatments (light and dark) for the glasshouse and Yanco field trials, while only the light treatment was applied for the Leeton field trial. For the glasshouse trial, the GI screen included 1385 and 1359 four-way MAGIC lines for the light and dark treatments, respectively. The Yanco field trial included 1063 four-way MAGIC lines for both dark and light treatments. For the Leeton field trial, only 232 the four-way MAGIC lines were screened for a GI. These lines were a selected subset from the lines also analyzed from the Yanco trial.

### Statistical analysis

Five analyses were conducted: the glasshouse light and dark screens, the Yanco 2009 field light and dark screens, and the Leeton 2010 screen (light only). Variation in the dormancy phenotype due to flowering time and germination conditions was accounted for in the analysis [36]. Summaries of the data are given in Additional file 2; note that for some analyses it was necessary to transform the GI because of strong non-normality of the index. Histograms of the GI for the five dormancy screenings are shown in Additional file 1. The raw indices are highly skewed and this was reflected in the residuals after initial fitting of the models described below.

A symbolic model for the GI that forms the basis of all analyses is:

$$ GI = Type + id + Block + Bench + Column. Row + Week + residual $$

where *GI* is the germination index, *Type* is a factor that separates out the MAGIC RILs from other lines in the trials, *Block*, *Bench*, *Row* and *Column* are factors that reflect the design in the growth phase, and *Week* is the design factor for the screening phase. Note that *Column.Row* is a factor consisting of combinations of the column and row position. The factor *id* indicates the line for each *GI*, be it a RIL or check line. All but *Type* are random effects in the model with an associated variance parameter.

For the Yanco field trial (both light and dark screening), plate position information was not recorded, but for the light screening there was information on blocking and row and column position for each week of screening, and these effects were included in the model. For the Leeton trial, an additional random effect due to column variation was included because it was evident in diagnostics [37].

The five analyses were conducted using asreml-R [38].

### Quantitative trait loci mapping

The QTL analysis was conducted using Multi-Parent Whole Genome Average Interval Mapping (MPWGAIM) [39], which utilizes the probability of inheriting founder alleles across the whole genome by simultaneously incorporating all information in the analysis, overcoming the need for repeated genome scans. To do so, a random effects working model is used in which all intervals are allowed to contain a possible QTL. A forward selection approach is used to select QTL. A likelihood ratio test of significance is conducted to decide if selection of a putative QTL is warranted or if selection should cease. An outlier statistic is used to select the most likely location for each QTL at the stage of the forward selection process. The approach allows for any non-genetic effects, such as experimental design terms, to be easily included in the base models. The five analyses described above formed the base models for the QTL analysis.

### Development of the near-isogenic lines

Based on the results of the QTL analysis, we identified five individual F_7_ RILs that were heterozygous (Yitpi allele versus any other allele) for the most tightly linked marker (CD920298) on 4AL whilst maximizing homozygosity for the remainder of the genome. These five individuals (numbers F1275, F0807, F0362, F1038, F1516) were then selfed to generate progeny which segregated according to Mendelian inheritance for dormancy. The progeny from these F_7_ derived lines were genotyped with the most tightly linked genetic marker, CD920298. These F_8_ lines were grown under glass in Canberra, Australia, in 2010. Each family was grown on a single bench in a randomized 6 × 8 layout. All plants were genotyped with the CD920298 marker and grouped according to the genotypes ‘+ +’, ‘ + −’, and ‘− −’. At physiological maturity two heads per plant were harvested and stored for subsequent dormancy screening. Several individuals with fixed QTL alleles were chosen in each HIF to generate sets of F_9_ NILs. The five sets of NILs were grown side by side together with the four parents. At 15, 25 and 35 DPA three seeds from three plants for each genotype class, along with the parents, were collected for RNA extraction.

### Transcriptomic analysis

#### Library generation and quality control and sequence alignment

RNA samples were prepared for sequencing using the Illumina TruSeq RNA Sample Prep Kit according to the manufacturer’s instructions. Each library was individually barcoded and sequenced on an Illumina HiSeq2000 platform using v2 chemistry. After initial quality control, the sequence datasets were pooled by technical replicate and three time points (15 DPA, 25 DPA and 35 DPA) into 84 pairs of paired-end read data files (168 files), in total consisting of 4,261,599,592 raw reads. Quality control and alignment steps are foundation elements to downstream SNP detection and differential expression. Given the size and complexity of the CSS that we used as reference set [11], two differing alignment approaches, Biokanga [40] and Gydle (Gydle Inc. Bioinformatics Service, Quebec City, Canada [41]), known to be able to analyze large reference sets, were used and results compared. Within Gydle, sequence reads were filtered using Nuclear (Gydle Inc.) and pair-end aligned to the wheat CSS contigs using Nuclear with moderately stringent parameters enabling two mismatches per read. Within Biokanga (version 3.4.5), reads were filtered to exclude exact duplicate reads by sequence and those reads whose sequence was not supported by at least two other overlapping reads to reduce the effect of potential sequence errors and PCR artifacts. In total, 1,467,808,800 reads were filtered, and the analysis was performed on the remaining 2,793,790,792 clean reads. Retained reads were pair-end aligned to the IWGSC CSS contigs. Alignment allowed at most two nucleic acid mismatches and only accepted reads with a single unique best alignment in each dataset. When aligned against the Chinese Spring survey sequences, 652,224,208 reads were accepted. Alignment yields were similar across all samples at around 20 % for both the Chinese Spring survey sequences. The second technical run showed approximately 40 % more reads and alignments compared with the first run, and 25 and 35 DPA displayed more reads aligned compared with 15 DPA. Transcriptome data have been deposited in the Sequence Read Archive on NCBI (accession number PRJNA278920).

#### SNP marker analysis

Biokanga SNP calling was performed on filtered reads of parent cultivars and near isogenic family progeny at individual time points. Putative SNPs identified for each time point were then combined. The SNP calling was aligned against the CSS contigs allowing at most two substitutions and unique alignments only. A custom R function was written to filter SNP results to identify candidate trait-linked SNPs based on allelic distribution observed in the parent cultivars and family progeny with QTL presence or absence. Applied criteria included: (1) the allele present in the founder Yitpi must be distinct from all other founders, including Baxter, Chara, and Westonia; (2) alleles observed in Yitpi must be either homozygous (≥95 % of reads in agreement for the allele) or absent; (3) four out of the five families must have the same SNP as a founder for that pool - Yitpi was the single founder for the QTL-positive pool, while Baxter, Chara, and Westonia were the founders for the QTL-negative pool; and (4) any candidate SNP marker must have had a minimum of two read coverage. Evidence for each candidate SNP was then manually inspected.

#### Differential analysis

Read counts of near isogenic family progenies were totaled for each transcript or contig and the resulting count matrix was normalized and analyzed for differential expression using the EdgeR [42] package in R version 3.0.1 [43]. The normalized data were fitted to a negative binomial generalized linear model incorporating covariates for experimental design parameters including family, time-point (15, 25 and 35 DPA) and the QTL status of the sample (positive or negative). A likelihood ratio test for identified contigs or transcripts with statistically significant QTL effects and resulting *P*-values were adjusted for multiple testing using the Hochberg false discovery rate adjustment approach. Contigs with an adjusted *P*-value smaller than 0.01 were considered differentially expressed.

### BAC sequencing and alignments

#### BAC screening and sequencing

A Chinese Spring wheat BAC library [13] was screened at the INRA-CNRGV [44] using the primers described in Additional file 6. In a first screening round, primers for the markers CD900298 and wsnp_Ex_c66324_64493429 were used and three BACs were identified (1816F24, 1836C17, 0251E06). A second round of screening was done by using a new set of primers for *PM19-A1*, which allowed us to identify four additional BACs of interest (1964H07, 0404N02, 1824C13, 1758D08). BAC DNA, isolated using the PhasePrep BAC DNA kit (Sigma-Aldrich, Sydney, Australia), was used in the preparation of paired-end (PE) and mate-pair (MP) libraries. For the paired-end libraries a single library for each BAC clone was prepared using the Nextera XT DNA library preparation kit (Illumina) following the manufacturer’s instructions. For the mate-pair libraries duplicate libraries were prepared on a pool of all BAC DNA using the Nextera Mate Pair Sample Preparation Kit (Illumina) with a lower size exclusion performed with Solid Phase Reversible Immobilization (SPRI) size selection kit (Beckman Coulter, NSW, Australia) to fragments greater than 1500 bp. All libraries were individually barcoded and sequenced on an Illumina HiSeq 2000 using v3 chemistry and Illumina MiSeq using v3 chemistry.

#### Sequence analysis and alignment

Fastq sequence files were filtered for high quality reads using the Nuclear software (Gydle Inc.) and assembled to individual BAC contigs and subsequent supercontigs using the Nuclear and Vision software (Gydle Inc.). BACs 1816 F24, 1836C17, 1964H07 were assembled into one supercontig; BACs 0404 N02 and 1824C13 were assembled into a second supercontig; and BACs 0251E06 and 1758D08 were assembled into a third. The GenBank accession numbers for the supercontigs are KP844896 (4AL), KP844897 (5BL) and KP844898 (5DL). Following assembly, the CSS contigs were aligned to the supercontigs (Additional file 7) using Nuclear and visualized in Vision, which allowed the supercontig to be mapped to chromosomes. Alignment of the Illumina paired-end re-sequencing from Yitpi, Chara and Westonia (Bio Platforms Australia) using Nuclear was used to confirm the SNP and INDEL identifications from targeted sequencing. The nucleotide sequences for *PM19-A1*, *PM19-A2* and *PM19-D3* from Yitpi and Chara have been deposited in GenBank with the following accession numbers: KP844883, KP844884, KP844885, KP844886, KP844887, KP844888.

For the phylogenetic analysis, nucleotide sequences of the seven Chinese Spring *PM19* genes and the *Arabidopsis* orthologue (At1g04560) identified by BLAST using the wheat PM19-A1 protein were aligned and a tree was generated using MEGA 6 [45]. The GenBank accession numbers for the wheat sequences used are KP844889 (*PM19-A1*), KP844890 (*PM19-A2*), KP844891 (*PM19-B1*), KP844892 (*PM19-B2*), KP844893 (*PM19-D1*), KP844894 (*PM19-D2*) and KP844895 (*PM19-D3*).

### Quantitative PCR analysis

For RNA extraction, frozen grains (or embryo and rest-of-grain samples for some experiments) were pulverized using a TissueLyser (Qiagen) and the total RNA was extracted [46]. RNA extractions were performed on three biological replicates of 10 grains (or embryos). The RNA was treated with DNase on mini RNeasy columns (Qiagen), and its quality was assessed on a NanoDrop 1000 Spectrophotometer (Thermo Scientific). A total of 2 μg of total RNA was then used to synthesize cDNA using SuperScript III (Invitrogen Life Sciences) following the supplier recommendations in 20 μl reactions. cDNA was diluted 50-fold and 10 μl was used in 20 μl PCR reactions with Platinum Taq (Invitrogen Life Sciences) and SYBR Green (Invitrogen). Specific primers were designed for the different genes studied and are listed in the Additional file 6. qPCR reactions were performed on a Corbett Rotor-Gene 6000 (Qiagen) and data was analyzed with Rotor-gene software using the comparative quantitation tool. The expression of *TaActin* [47] was used as an internal control to normalize gene expression.

For ABA treatment, triplicate sets of 20 dormant (freshly harvested) or after-ripened (showing 100 % germination) grains were placed on 90-mm Petri dishes containing one 90 mm Whatman 598 filter paper and 5 mL of water or 10^−4^ M ABA. Embryos were dissected after 24 h of incubation in darkness.

### Generation of transgenic plants

Hairpin RNAi constructs targeting *PM19-A1* and *A2* were made by inserting the whole coding *PM19-A2* sequence in both orientations into the hairpin RNAi vector pStarling [48]. The hairpin RNAi constructs were subcloned into the NotI site of the binary vector pWBVec8 [49]. Biolistic transformation of wheat was performed [50, 51]. Immature wheat embryos, following 3–5 days of preculture, were bombarded, using gold as micro-carrier, with plasmids containing genes of interest and pCMneoSTLS2, which encodes an intron-containing neomycin phosphostransferase gene regulated by a CaMV 35S promoter [52]. Wheat callus was cultured under selection before shoot and root formation was initiated [53]. T_0_ plants were transferred to soil, screened for transgene integration, and self-fertilized to generate T_1_ progeny. T_2_ homozygous populations from four independently transformed RNAi lines along with their corresponding null segregant lines (which were derived from the same T_0_ line) were isolated. T_3_ grains from homozygous T_2_ plants (about 25 plants per genotype) grown in naturally lit phytotron glasshouses with air temperature set at 17 °C/9 °C day/night cycle were harvested at maturity for dormancy test and expression studies.

## Additional files

Additional file 1: Figure S1. Histograms of the germination index for the five dormancy screenings. Additional file 2: Table S1. Summaries for the dormancy data. Additional file 3: Table S2. QTL results for all sites. Additional file 4: Table S3. CSS contigs differentially expressed between pools A and B. Additional file 5: Table S4. Detected SNPs in the pool analysis using CSS contigs. Additional file 6: Table S5. Primers used in this work. Additional file 7: Table S6. CSS contigs mapped to the different supercontigs.

## Abbreviations

ABA: abscisic acid
ABRE: ABA-response element
BAC: bacterial artificial chromosome
bp: base pair
CSS: Chromosome Survey Sequence
DPA: days post-anthesis
EST: expressed sequence tag
GI: Germination Index
HIF: heterogeneous inbred family
IWGSC: International Wheat Genome Sequencing Consortium
MAGIC: Multi-parent Advanced Generation Inter-Cross
NIL: near-isogenic line
PCR: polymerase chain reaction
PHS: pre-harvest sprouting
qPCR: quantitative PCR
QTL: quantitative trait locus
RNAi: RNA interference
SNP: single nucleotide polymorphism
