# Supplementary figures and images for: Transcriptomic analysis of wheat near-isogenic lines identifies PM19-A1 and A2 as candidates for a major dormancy QTL
Source: Genome Biol. 2015 May 12;16(1):93. doi: 10.1186/s13059-015-0665-6 (PMC4443510; doi:10.1186/s13059-015-0665-6)

## Slide 1
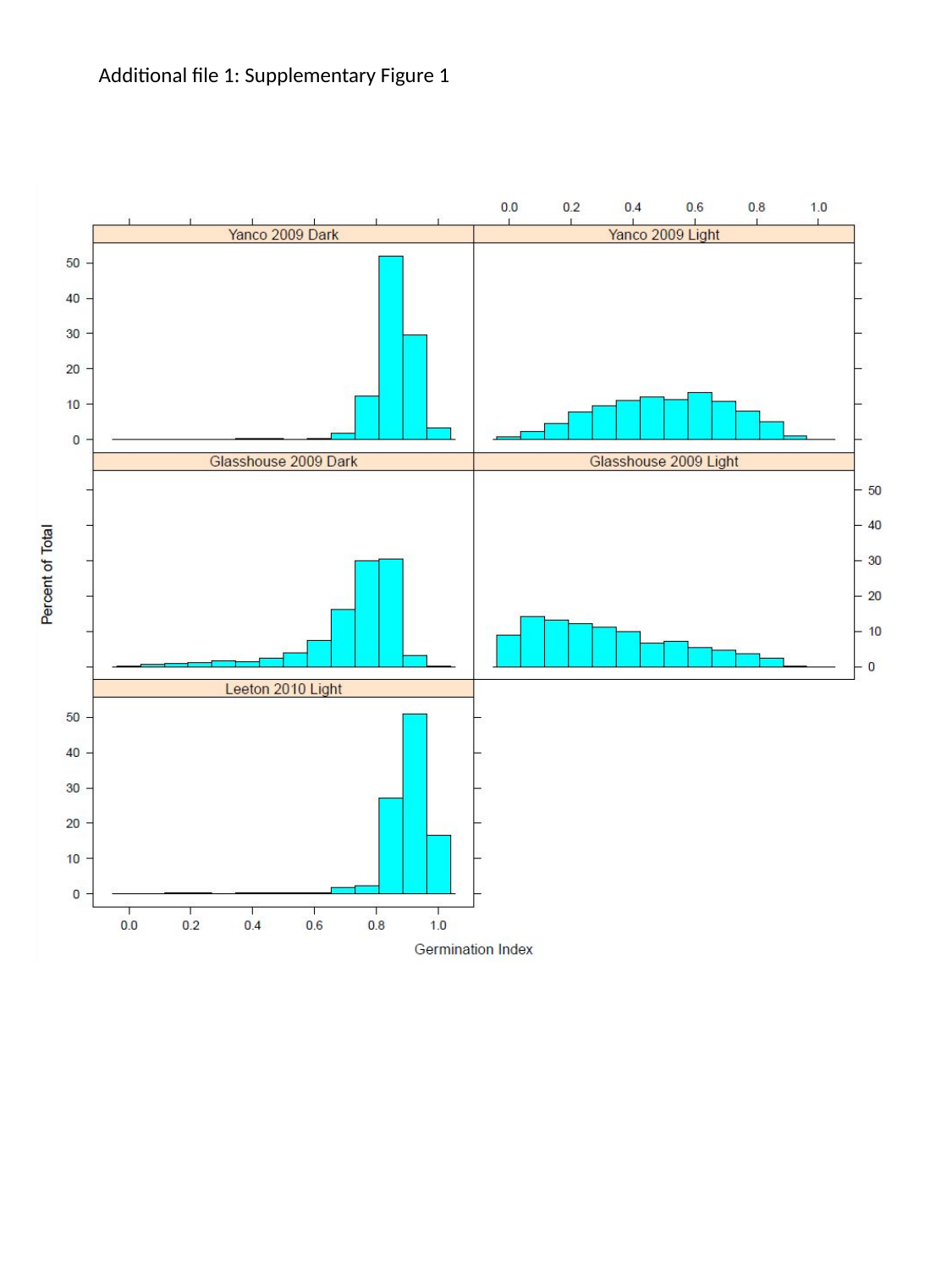

Additional file 1: Supplementary Figure 1

Supplement: Additional file 1: Figure S1. — Histograms of the germination index for the five dormancy screenings. [file 13059_2015_665_MOESM1_ESM.pptx]
